# Orthopaedic surgeons display a positive outlook towards artificial intelligence: A survey among members of the AGA Society for Arthroscopy and Joint Surgery

**DOI:** 10.1002/jeo2.12080

**Published:** 2024-07-06

**Authors:** Marco‐Christopher Rupp, Lukas B. Moser, Silvan Hess, Peter Angele, Matthias Aurich, Felix Dyrna, Stefan Nehrer, Markus Neubauer, Johannes Pawelczyk, Kaywan Izadpanah, Johannes Zellner, Philipp Niemeyer, AGA‐Komitee Innovation und Translation

**Affiliations:** ^1^ Sektion Sportorthopädie, Klinikum rechts der Isar Technische Universität München Munich Germany; ^2^ Steadman Philippon Research Institute Vail Colorado USA; ^3^ Klinische Abteilung für Orthopädie und Traumatologie Universitätsklinikum Krems Krems an der Donau Austria; ^4^ Zentrum für Regenerative Medizin Universität für Weiterbildung Krems Krems an der Donau Austria; ^5^ Sporthopaedicum Regensburg Germany; ^6^ Universitätsklinik für Orthopädische Chirurgie und Traumatologie, Inselspital Bern Switzerland; ^7^ Klinik für Unfall‐ und Wiederherstellungschirurgie Universitätsklinikum Regensburg Regensburg Germany; ^8^ Universitätsklinikum Halle (Saale) Halle Germany; ^9^ Gelenkzentrum Leipzig Leipzig Germany; ^10^ Fakultät für Gesundheit und Medizin Universität für Weiterbildung Krems Krems an der Donau Austria; ^11^ Klinik für Orthopädie und Unfallchirurgie, Universitätsklinikum Freiburg, Medizinische Fakultät Albert‐Ludwigs‐Universität Freiburg Freiburg Germany; ^12^ OCM – Orthopädische Chirurgie München Munich Germany; ^13^ Albert‐Ludwigs‐University Freiburg Germany

**Keywords:** artificial intelligence, attitude of health personnel, machine learning, orthopaedics, survey

## Abstract

**Purpose:**

The purpose of this study was to evaluate the perspective of orthopaedic surgeons on the impact of artificial intelligence (AI) and to evaluate the influence of experience, workplace setting and familiarity with digital solutions on views on AI.

**Methods:**

Orthopaedic surgeons of the AGA Society for Arthroscopy and Joint Surgery were invited to participate in an online, cross‐sectional survey designed to gather information on professional background, subjective AI knowledge, opinion on the future impact of AI, openness towards different applications of AI, and perceived advantages and disadvantages of AI. Subgroup analyses were performed to examine the influence of experience, workplace setting and openness towards digital solutions on perspectives towards AI.

**Results:**

Overall, 360 orthopaedic surgeons participated. The majority indicated average (43.6%) or rudimentary (38.1%) AI knowledge. Most (54.5%) expected AI to substantially influence orthopaedics within 5–10 years, predominantly as a complementary tool (91.1%). Preoperative planning (83.8%) was identified as the most likely clinical use case. A lack of consensus was observed regarding acceptable error levels. Time savings in preoperative planning (62.5%) and improved documentation (81%) were identified as notable advantages while declining skills of the next generation (64.5%) were rated as the most substantial drawback. There were significant differences in subjective AI knowledge depending on participants' experience (*p* = 0.021) and familiarity with digital solutions (*p* < 0.001), acceptable error levels depending on workplace setting (*p* = 0.004), and prediction of AI impact depending on familiarity with digital solutions (*p* < 0.001).

**Conclusion:**

The majority of orthopaedic surgeons in this survey anticipated a notable positive impact of AI on their field, primarily as an assistive technology. A lack of consensus on acceptable error levels of AI and concerns about declining skills among future surgeons were observed.

**Level of Evidence:**

Level IV, cross‐sectional study.

**Abbreviations**
AGA: German‐Speaking Society for Arthroscopy and Joint Surgery
AI: artificial intelligence
LLM: large language model
ML: machine learning

## INTRODUCTION

With its enormous potential in healthcare, artificial intelligence (AI) is playing an increasingly important role in research and clinical care [19, 27, 35, 49]. Defined as the ability of machines to solve tasks that traditionally require human intelligence [16], AI integration into clinical practice has been pioneered in data‐heavy specialties, such as radiology, oncology, or pathology [30], whereas the application in more manual specialties such as orthopaedics has been more reluctant [5, 30].

While the complexity and rapidness in the development of AI technologies may represent a barrier for full‐time clinicians aiming to reach and maintain an up‐to‐date technical understanding, several applications of AI specific to orthopaedic surgery have been developed, including preoperative risk stratification [19, 31, 35], outcome prediction [27], diagnosis and preoperative planning [45, 46, 49], augmentation of postoperative rehabilitation [4, 11], automated administration [26, 51] and patient information [18].

The appeal of utilising AI across a broad spectrum of clinical applications in orthopaedics varies across use cases. This includes the capacity to manage extensive volumes of interdependent data, offer clinical insights [27], uncover previously unknown relationships within large datasets [32], ensure superior accuracy or consistency [44] and even surpass human experts (e.g., in terms of speed) in certain clinical tasks [43].

The role of AI in various clinical use cases is diverse, encompassing the augmentation of orthopaedic experts, alleviating the administrative burden, and potentially even replacing orthopaedic or administrative personnel in specific subtasks. In the context of the swift and controversial deployment of this exponentially powerful technology, the potential benefits and drawbacks of AI are extensively debated within professional societies [36, 39, 41, 48].

Considering the inevitability of contact with this technology due to its rapid consumer‐facing deployment and increasing public awareness [37], the sentiment of medical professionals towards AI is pivotal [23]. It may play a crucial role in determining whether the organic integration of AI will elevate physician's capabilities or whether lower degrees of acceptance will result in the perception of AI as an adversary [24]. While other specialities have assessed this sentiment already [36, 39, 41, 48], there is a clear knowledge gap regarding the perspectives of orthopaedic surgeons towards AI.

Thus, the primary aim of this study was to assess the perspectives of orthopaedic surgeons on the impact of AI in orthopaedic surgery. The secondary objective was to explore whether the overall attitude towards AI in orthopaedic surgery would vary based on experience, workplace setting, or general familiarity with digital solutions in subgroup analyses. It was hypothesised that a mixed attitude towards AI in orthopaedic surgery would emerge, with results depending on the above‐mentioned variables.

## METHODS

This was a cross‐sectional survey‐based study, which was approved by the board of the AGA Society for Arthroscopy and Joint Surgery prior to data collection. The survey, designed by the members of the committee “Innovation und Translation” of the AGA Society for Arthroscopy and Joint Surgery, was distributed via an online service provider (SurveyMonkey Inc.) among full members of the AGA Society for Arthroscopy and Joint Surgery, that is, residents and board‐certified surgeons. The AGA comprises members in Germany, Switzerland, and Austria, and for the purpose of this study, only medically licensed members were included. Following an initial invitation via email, eligible participants who did not complete the questionnaire were contacted once again after 7 days. Participation in the survey was voluntary. Refusal to participate in the survey had no negative consequences for members. The survey accepted new participants for a total of 28 days starting on January 2023, and all data were collected anonymously.

## SURVEY DESIGN

The survey comprised a total of 14 questions and was designed according to previously published surveys [36, 39, 48] in a plenary discussion among the members of the committee “Innovation und Translation” of the AGA Society for Arthroscopy and Joint Surgery; the questionnaire was designed and distributed in German. An English translation of the complete questionnaire is available in Table S1.

Questions 1–4 were designed to gather information regarding participants' professional background regarding work experience (<5, 5–10, 11–15, >15 years), practice focus (predominantly non‐operative, predominantly arthroscopic surgery, predominantly open surgery), workplace setting (university hospital, academic teaching hospital, peripheral hospital, private practice) and familiarity with the use of digital solutions not related to AI (a comprehensive list of applications potentially used in clinical practice). Question 5 rated participants' subjective knowledge about AI; questions 6–8 assessed the participants' opinion on the time frame, specific areas, and general impact of AI on orthopaedic surgery; questions 8–9 assessed the participants' openness towards different applications of AI in clinical practice as well as the acceptable level of error; questions 12–13 analysed participants' perceptions of specific advantages and disadvantages of AI in orthopaedic surgery. Depending on the question, both single‐choice as well as multiple‐choice question designs were employed. Based on the software employed, not completing a question was allowed, and incomplete datasets were included in the final analysis.

## SUBGROUP ANALYSES

Subgroup analyses were conducted on a subset of the AI‐specific inquiries based on experience, practice focus, workplace setting, and familiarity with digital solutions, which was operationally defined as the utilisation of at least one digital solution within the participant's practice. The limited sample size imposed constraints on the number of subgroup analyses, as an excessive number of comparisons on a single dataset increases the risk of type 1 errors. Consequently, meaningful subgroup comparisons were identified *a priori* and included in the assessment of the study's secondary hypothesis. These encompassed subjective knowledge of AI in medicine, the anticipated time frame for the noticeable impact of AI on orthopaedic surgery, the overall influence of AI on the field of orthopaedic surgery, and the acceptable error level for clinical AI applications.

## STATISTICAL ANALYSIS

Statistical analysis was performed using SPSS software version 26.0 (IBM‐SPSS). Categorical variables were reported as counts and percentages. Categorical variables were compared using the binary Fisher's exact test or the Chi‐square test as statistically appropriate. The level of significance was set at *p* < 0.05. An a priori power analysis was not performed, as the number of participants limited the size of the available dataset.

## RESULTS

**Figure 1:**
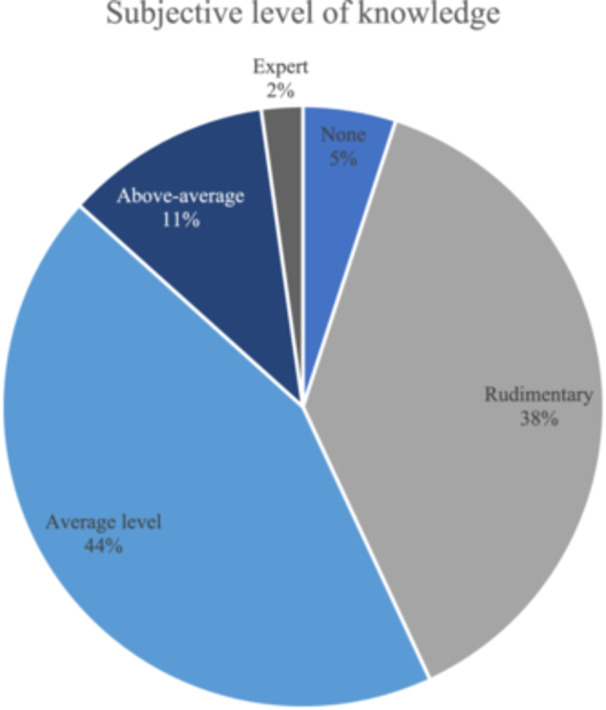
Self‐estimated artificial intelligence (AI) knowledge. Pie chart illustrating the subjective rating of knowledge of AI in medicine among 360 participants.

**Figure 2:**
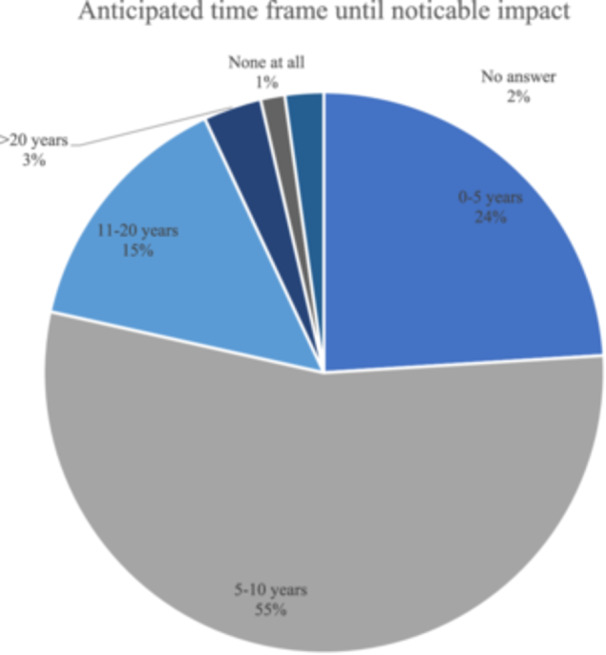
Anticipated time frame until noticeable impact. Pie chart illustrating respondents' anticipated time frame until AI exhibits a noticeable impact on orthopaedic surgery among 360 participants.

A total of 360 orthopaedic surgeons participated in this survey (4159 surgeons contacted, 8.7% response rate), with the majority of participants working in private practice and with a strong focus on arthroscopic surgery. Detailed demographic data are available in Table 1. Most respondents indicated average knowledge about AI, as illustrated in Figure 1. The majority of participants believed AI would have a noticeable impact on orthopaedic surgery within the next 5–10 years (cf. Figure 2), and most participants expected AI to be an auxiliary tool in specific areas (*n* = 328, 91.1%), rather than replacing orthopaedic surgeons in central capacities of the profession (*n* = 25, 6.9%) or not influencing the job profile at all (*n* = 6, 1.7%).

**Table 1:** Workplace, practice focus, experience, and familiarity with digital solutions.

	Total number	Percentage
**Workplace**		
University hospital	66	18.4
Academic teaching hospital	103	28.7
Peripheral hospital	79	22.0
Private practice	146	40.7
Not specified	7	2.0
**Practice focus**		
Mostly nonoperative	20	5.6
Mostly surgical (arthroscopic)	199	55.4
Mostly surgical (open surgery)	111	30.9
Not specified	29	8.1
**Experience**		
<5 years	41	11.4
5–10 years	64	17.8
11–15 years	61	17.0
>15 years	193	53.8
Not specified	0	0.0
**Familiarity with digital solutions**		
Use of ≥1 digital solutions	302	83.9
Use of <1 digital solutions	58	16.1
Not specified	0	0.0

*Note*: Stratification of the participants by workplace setting, practice focus, work experience, and general familiarity with digital solutions. Categorical variables are presented as counts and percentages.

**Figure 3:**
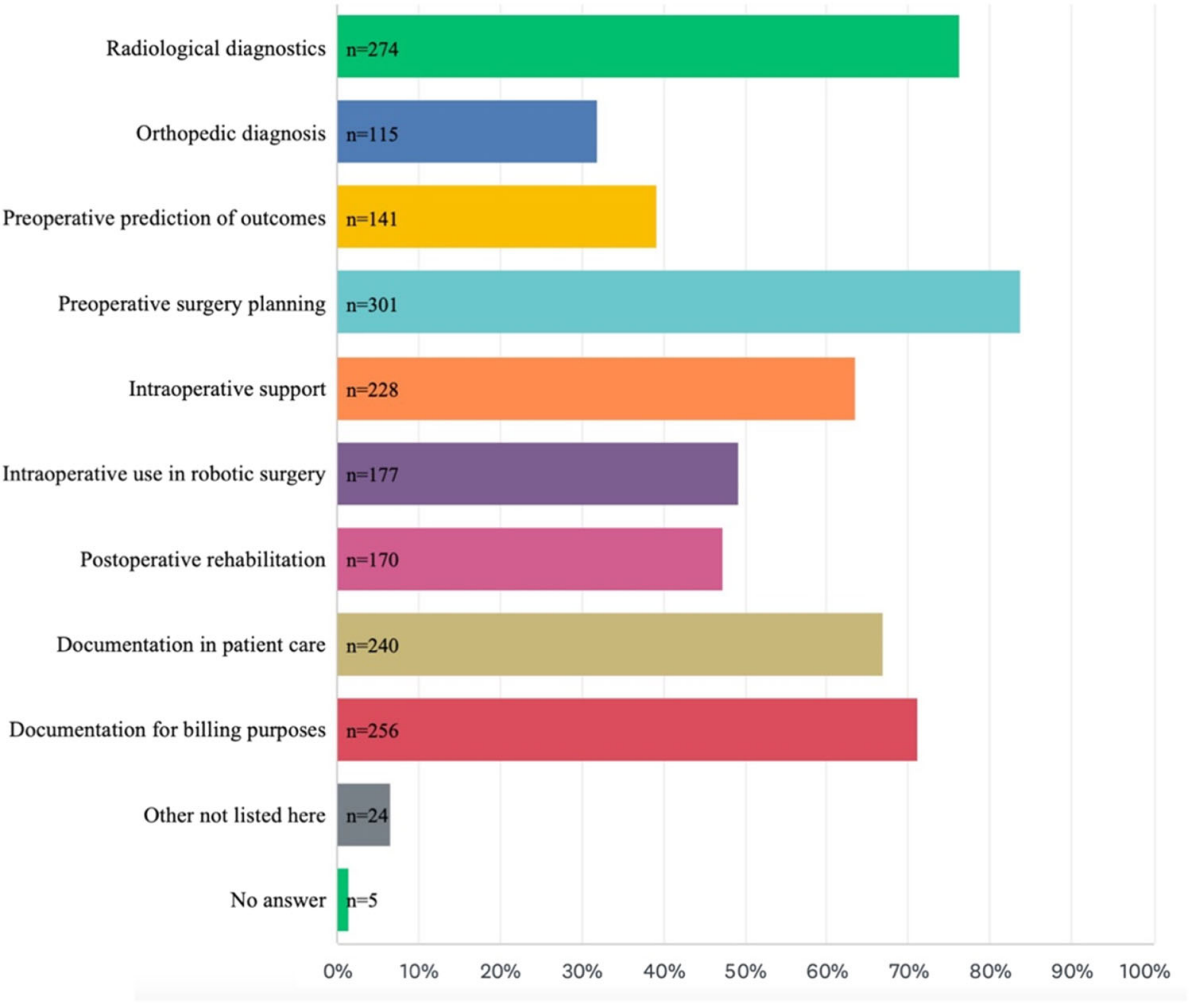
Areas of clinical use. Anticipated areas of clinical use for artificial intelligence in orthopaedics (multiple‐choice). Bar length correlates with the frequency of answers chosen in this multiple‐choice question design.

The most likely areas of clinical use of AI are detailed in Figure 3, including preoperative surgical planning, radiological diagnosis and documentation for billing purposes. Furthermore, openness towards different hypothetical applications of AI in clinical practice was assessed and is summarised in Table 2. Regarding the acceptable error level of AI, 14.7% (*n* = 52), 22.8% (*n* = 82), 22.6% (*n* = 81), and 27.9% (*n* = 100) of the participants required the average acceptable competence to be on the level of a resident physician, board‐certified surgeon, attending physician, or recognised expert in the field, respectively.

**Table 2:** Applications of artificial intelligence (AI).

Clinical use case	Total	%
A patient's medical history is collected digitally before a clinic visit and analysed using AI. A data‐sheet with the most important facts and potential diagnoses is provided to a medical specialist in advance to increase the efficiency of the doctor's visit.	210	58.6
Radiological findings of a patient are analysed with AI. An orthopaedic specialist reviews both the image and the results of AI and makes a diagnosis based on them.	271	75.7
All relevant preoperative data of a patient are analysed with AI and provided a therapy suggestion. Based on this information, a specialist physician makes a therapy decision.	148	41.3
After the treatment intervention, the patient is provided with an AI‐based messaging system. This system notifies the physician if the patient has complications or if the rehabilitation process seems abnormal.	230	64.3

*Note*: Different hypothetical applications of AI in clinical practice were presented and the openness to using these solutions was evaluated.

**Figure 4:**
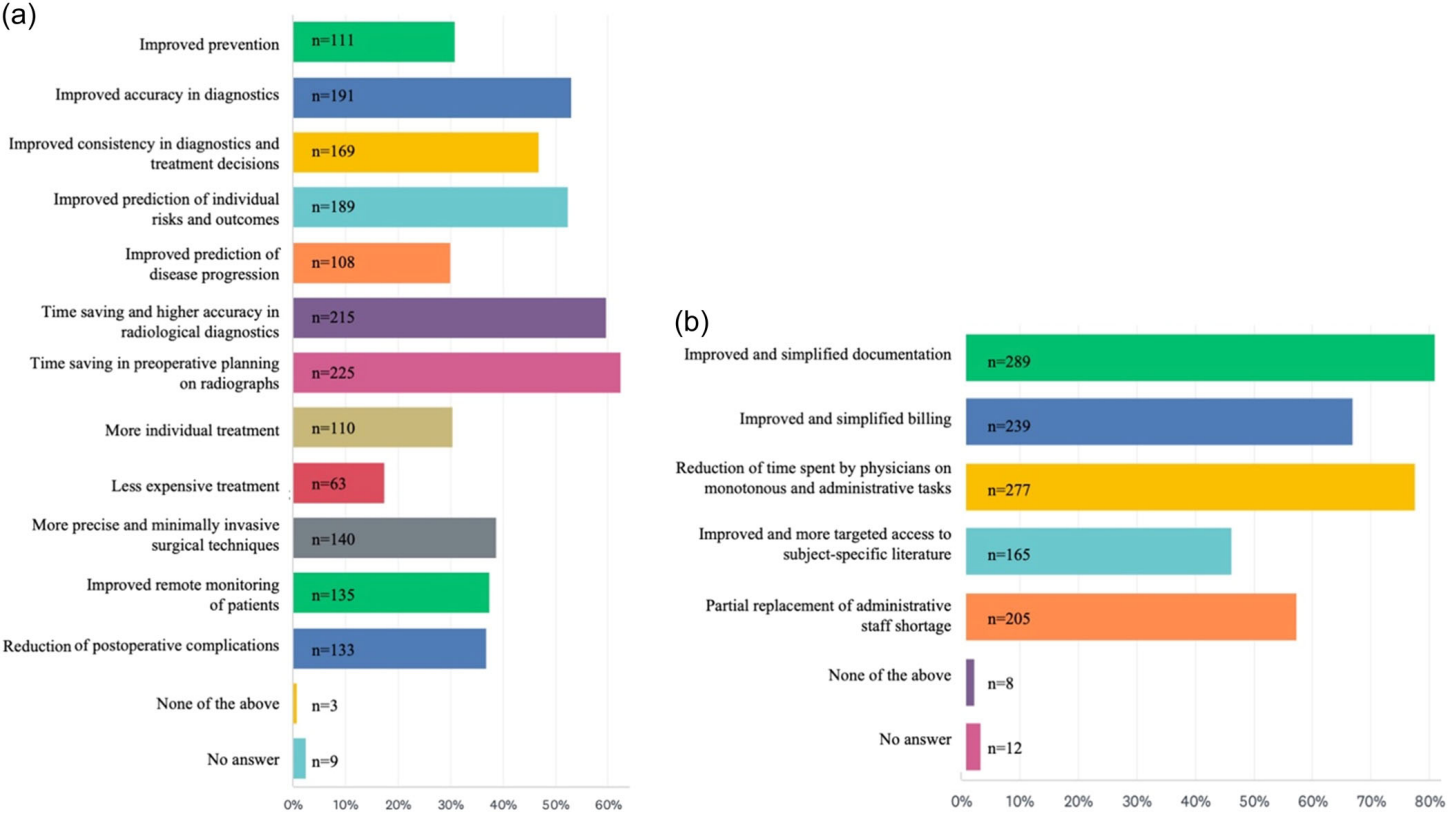
Benefitial use cases. Use cases of greatest potential benefit for the use of artificial intelligence in (a) direct patient care and (b) activities outside of direct patient care in orthopaedics. Bar length correlates with the frequency of answers chosen in this multiple‐choice question design.

**Figure 5:**
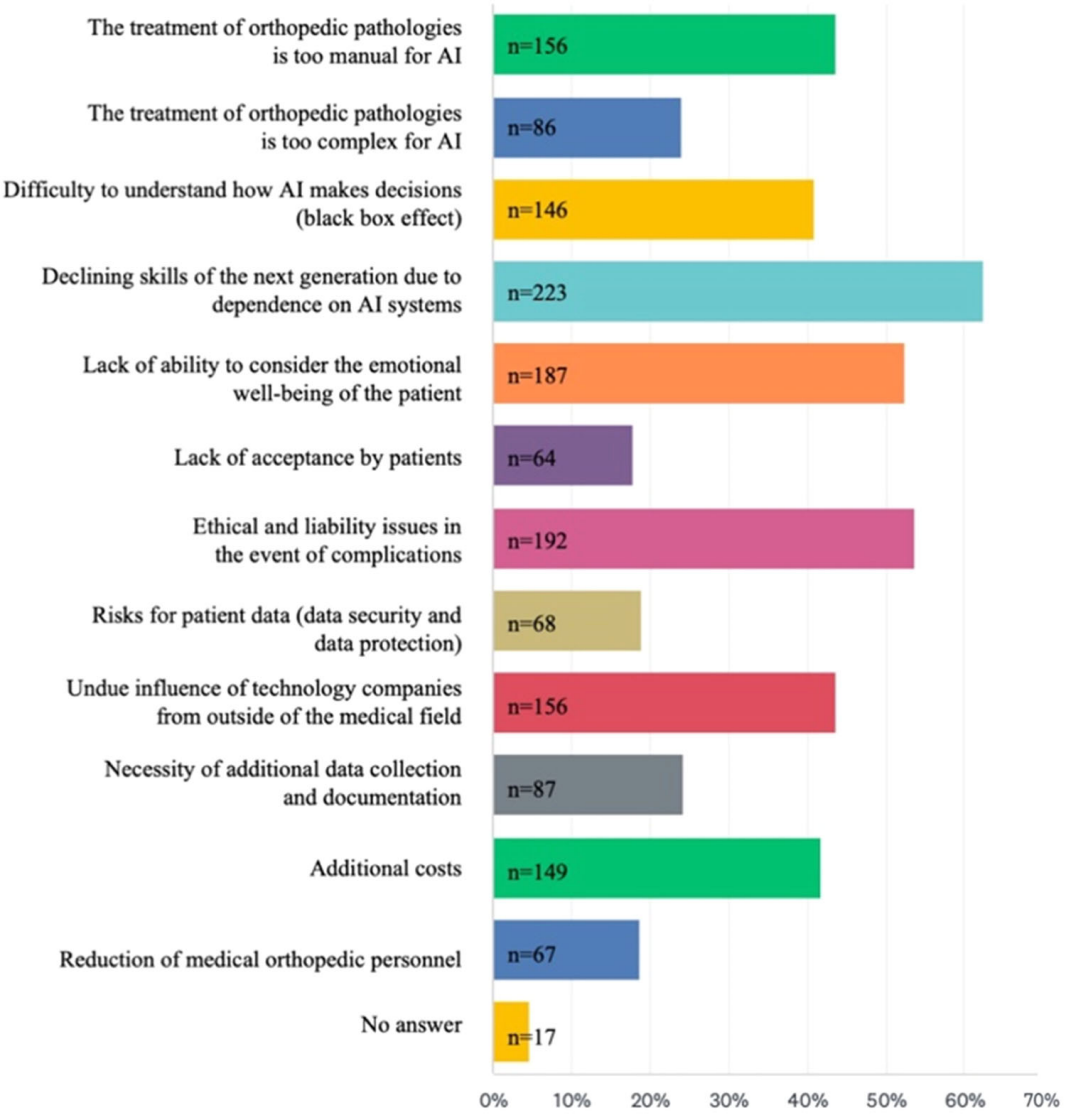
Disadvantages of artificial intelligence (AI). Greatest potential drawbacks for the use of artificial intelligence in orthopaedics. Bar length correlates with the frequency of answers chosen in this multiple‐choice question design.

In clinical care, time savings in preoperative planning and radiological diagnostics, as well as improved diagnostic accuracy and improved prediction of individual risks and outcomes, were identified as the use cases of greatest potential benefit (cf. Figure 4a). In regard to activities outside of direct patient care, improved and simplified documentation and billing, as well as physician involvement in monotonous and administrative tasks, were identified as the use cases of greatest potential benefit (cf. Figure 4b). Declining skills among the next generation of orthopaedic surgeons due to overreliance on AI and ethico‐legal concerns were rated as the most substantial drawbacks, while a reduction of orthopaedic physician personnel or a lack of acceptance by patients were the least commonly identified drawbacks, as explained in Figure 5.

### Subgroup analyses

Depending on professional background, significant differences in subjective AI knowledge were observed (*p* = 0.021). As such, the largest percentage (12%) of participants indicating ‘no knowledge’, was found among respondents with <5 years of experience, while the highest percentage (3.1%) indicating ‘expert knowledge’, was found among respondents with >15 years of experience (cf. Table S2).

Depending on the practice setting, significant differences in regard to the acceptable level of error of AI applications were observed (*p* = 0.004). While orthopaedic surgeons working in academic teaching hospitals were the subgroup most commonly requiring only a level of error corresponding to a resident physician (22.7%), respondents working in university hospitals most commonly required a level of error corresponding to a board‐certified orthopaedic surgeon (33.9%). Furthermore, respondents working in a non‐academic setting were found to require a level of error corresponding to a recognised expert in the field (36.2%), with a similar trend among surgeons working in private practice (29.0%) (Table S3).

Familiarity with digital applications was highly predictive of subjective AI knowledge (*p* < 0.001) and prediction of the noticeable impact of AI on orthopaedic surgery (*p* < 0.001). Among surgeons familiar with digital applications, the level of subjective AI knowledge was higher, and the anticipated time frame until AI would have a noticeable impact on orthopaedic surgery was shorter (cf. Table S4).

## DISCUSSION

The key finding of this study was that most orthopaedic surgeons anticipate that AI will significantly influence orthopaedic surgery in the near future, mainly serving as an assistive technology in preoperative planning and documentation. There was a lack of consensus on the acceptable error level of AI, and there were concerns about declining skills among the next generation of surgeons due to overreliance on AI systems. These results also revealed significant differences in attitude towards AI, based on experience, workplace setting, and familiarity with digital solutions.

While the rapidly increasing technological advancement of AI has affected other medical specialities such as radiology, histology and cardiology earlier, more directly, and more substantially [3], a growing body of evidence evaluating the clinical application of AI solutions in orthopaedics has recently been generated. Notably, for surgical disciplines, back in 2016, a survey among 1634 experts on AI forecasted that AI may ‘work as a surgeon’ as early as 2053 [14]. Other forecasts from this survey, such as AI's ability to translate languages by 2024 and write school essays by 2026 [14], have recently been achieved and even outpaced due to the rapid development of large language models (LLM). Due to the growing impact of AI on the entire continuum of care in orthopaedics, less central tasks in the orthopaedic profession are likely going to be impacted earlier. Consequently, moving forward, the acceptance and attitude of key stakeholders will be pivotal in determining whether an organic integration of AI will enhance orthopaedic surgeons' capabilities or be perceived as an adversary [24].

In regard to the expected time frame, the majority of orthopaedic surgeons predicted a notable influence in the next 5–10 years, with less than 2% predicting no impact at all. This is similar to the perception of a mixed group of non‐orthopaedic surgeons, of which 47.2% predicted an impact in the next 5–10 years [8, 39], while trauma and emergency medicine surgeons rated the impact of AI as 3.88 of 5 in the next 5 years [8].

The vast majority (91.1%) of orthopaedic surgeons predicted that AI would only become an auxiliary tool and not replace orthopaedic surgeons. These findings contrast with survey studies conducted among radiologists, which revealed that the majority of the participants expected their job description to change significantly within the next 5–10 years (31.6%) or 10–20 years (61.1%) [10]. Up to 12.0% of the radiologists even predicted that AI would *replace* their profession [38], and up to 67.7% predicted that a reduced number of radiologists would be needed [13]. This difference may be due to the perceived manual nature of the orthopaedic profession, which will require significant developments not only in AI but also in robotics [15].

In regard to subjective knowledge of AI, most respondents rated their knowledge as average (44%), while there was a substantial portion of the surgeons who rated their level as rudimentary (38%). This distribution is comparable to other clinical specialities, such as general surgery, dermatology, or ophthalmology [39, 41], while in more technical specialities, such as radiology, the perceived knowledge was rated as intermediate or advanced in up to 39% of the respondents [17]. Crucially, a lack of basic familiarity with AI may have negative consequences for scientists and clinicians due to the increasing importance of this technology in many key aspects of healthcare.

The participants in this study identified preoperative planning and radiographic diagnosis, as well as documentation, as the most likely use cases for AI in orthopaedic surgery. In general, this aligns well with the areas of clinical orthopaedic use currently explored in various feasibility studies, which can broadly be classified as administrative support, risk stratification, or outcome prediction for orthopaedic procedures and musculoskeletal imaging diagnostics [32]. As such, ML‐based prediction models have not only been successfully explored for their ability to predict clinical outcomes following hip arthroscopy [28], cartilage restoration [40], and shoulder arthroplasty [19] but also for the ability to predict injury [21], which approximately 40% of orthopaedic surgeons identified as likely use cases of clinical application. In an effort to lower the administrative burden, ML algorithms have been leveraged to automatically generate billing codes from operative reports [25], as well as to predict length of stay and inpatient cost [19]. Accordingly, in this study, up to 81% of orthopaedic surgeons identified simplified and less time‐consuming documentation as the use cases with the greatest potential benefit of AI; findings are in line with previous investigations in other specialities [41, 50]. Furthermore, there is substantial evidence that tools such as convolutional neural networks, as well as advanced deep learning networks, can be employed in musculoskeletal radiology in use cases such as radiologic diagnosis [6, 12], severity grading [34], implant recognition [20, 22], implant size prediction [29], and analysis of complex radiographic indices and measurements [7, 43]. In line with these developments, the majority of orthopaedic surgeons (75.7%) in the present study signalled their openness towards a clinical workflow, including diagnostic imaging, which was preanalysed by an AI. However, potentially attributable to the substantial media attention towards the extensive progress in natural language understanding and conversational ability of LLMs during the time of the survey [1, 9, 47], the majority of the orthopaedic surgeons could also envision working with an AI summarising a patient case and a providing a differential diagnosis as well as an AI‐based alert system during rehabilitation. In line with the expectations, automated postoperative messaging systems during rehabilitation have already been demonstrated to result in increased satisfaction and improvement in clinical endpoints [2, 42].

However, there was substantial heterogeneity in regards to the acceptable level of clinical accuracy of AI solutions, with approximately equal distribution between board‐certified surgeons, attending physicians, and recognised experts in the field, while up to 14.6% of the participants accepted a resident's level of error. In other disciplines, the level of acceptable error was of similar distribution for screening for disease; for diagnostic decisions, up to 64% of the surgeons required a level of experience >15 years or greater [39, 41].

There also remain substantial concerns with the integration of AI into orthopaedics, with the majority of the participants seeing the dependence of the next generation of orthopaedic surgeons on AI systems and ethical and legal concerns as the most substantial drawbacks. This is in accordance with previous studies identifying concerns over legal responsibility as a major barrier to the clinical adoption of AI [33, 41, 50]. Moving forward, to ensure successful clinical adoption, more comprehensive regulation will be necessary to provide guidelines on this concern for orthopaedic surgeons [33]. Interestingly, in the present study, there were only limited concerns regarding patient acceptance of AI in orthopaedic surgery, most likely due to the majority of respondents believing that AI would act as an augmentation to doctors rather than a replacement.

This study has several notable limitations. Inherent with the survey study design, only a fraction of members of the AGA Society for Arthroscopy and Joint Surgery participated in this survey, indicating a potential non‐response bias; however, the participating surgeons represented the member structure of the AGA well. Furthermore, efforts were made to reduce possible sampling bias by including surgeons across various professional backgrounds. By including a ‘no answer’ option, efforts were made to reduce possible response bias. Additionally, demand characteristics bias may have influenced results. The external geographic validity of these results may be limited. Finally, this survey can only capture current dispositions, given rapid advancements in the field of AI.

## CONCLUSION

The majority of orthopaedic surgeons in this survey anticipated a notable positive impact of AI on their field, primarily as an assistive technology. A lack of consensus on acceptable error levels of AI and concerns about declining skills among future surgeons were observed.

## AUTHOR CONTRIBUTIONS

All listed authors have contributed substantially to this work. Marco‐Christopher Rupp, Lukas B. Moser, Silvan Hess, Peter Angele, Matthias Aurich, Felix Dyrna, Stefan Nehrer, Markus Neubauer, Johannes Zellner and Philipp Niemeyer engaged in the study conception and design. Marco‐Christopher Rupp designed the survey questionnaire, which was critically revised by all authors. Marco‐Christopher Rupp performed the data collection and analysis. Marco‐Christopher Rupp performed the data interpretation. Marco‐Christopher Rupp and Lukas B. Moser drafted the manuscript. Marco‐Christopher Rupp drafted the figures and tables, and performed the literature research. All authors critically revised the manuscript.

## CONFLICT OF INTEREST STATEMENT

Peter Angele is a consultant for Aesculap/TETEC and Arthrex. The remaining authors declare no conflict of interest.

## ETHICS STATEMENT

Not applicable.

## Supporting information

Supporting information.

Supporting information.

Supporting information.

Supporting information.

## Data Availability

Datasets used and/or analysed during the current study are available from the corresponding author upon reasonable request.
