# Supplementary material for: Orthopaedic surgeons display a positive outlook towards artificial intelligence: A survey among members of the AGA Society for Arthroscopy and Joint Surgery
Source: J Exp Orthop. 2024 Jul 6;11(3):e12080. doi: 10.1002/jeo2.12080 (PMC11227606; doi:10.1002/jeo2.12080)
Supplement: Supplementary file 1 — Supporting information. [file JEO2-11-e12080-s003.docx]

| ***Survey Questionnaire*** |
| --- |
| **1. Practice setting**   - University Hospital - Academic teaching hospital - Non-academic hospital - Private practice - Not specified |
| **2. Practice focus**   - Predominantly non-operative - Predominantly surgical (arthroscopic operations) - Predominantly surgical (open operations) - Not specified |
| **3. Work experience**   - <5 years - 5-10 years - 11-15 years - ≥15 years - Not specified |
| **4. Do you currently already use digital solutions, which are not based on artificial intelligence, in your daily clinical routine? (Multiple choice)**   - Telemedicine offer - Digital anamnesis software - Digital reconnaissance software - Augmented reality (surgery, consultation) - App for inpatient care of patients - Digital planning program - Operating room robot - Digital recording of clinical scores and satisfaction - App for postoperative care - App for digital physiotherapy - Other - None |
| **5. How would you rate your knowledge of AI in medicine in general?**   - Expert knowledge - Above average knowledge - Average knowledge - Rudimentary knowledge - No knowledge |
| **6. Do you think AI will have a noticeable impact on joint surgery in the future, and if so, how long will it take?**   - Never - 0-5 years - 5-10 years - 11-20 years - > 20 years - No answer |
| **7. If you think artificial intelligence could have an impact on joint surgery, in which area do you see AI being used (Multiple choice)?**   - Radiological diagnostics - Orthopedic diagnosis - Preoperative prediction of outcomes - Preoperative surgery planning - Intraoperative support - Intraoperative use in robotic surgery - Postoperative rehabilitation - Documentation in patient care - Documentation for billing purposes - Other not listed here - No answer |
| **8. How do you think AI will impact the orthopedic/joint surgery profession in the next 25 years?**   - AI will not influence the job profile - AI becomes a complementary tool in certain areas of the profession - AI will replace orthopedic/joint surgeons in key activities - No answer |
| **9. Would you consider using the following AI-based clinical workflows? (Multiple choice)**   - A patient's medical history is collected digitally before a clinic visit and analyzed using artificial intelligence. A data sheet with the most important facts and potential diagnoses is provided to a medical specialist (m/f/d) in advance to increase the efficiency of the doctor's visit. - Radiological findings of a patient are analyzed with artificial intelligence. An orthopedic specialist (m/f/d) reviews both the image and the results of the artificial intelligence and makes a diagnosis based on them. - All relevant preoperative data of a patient are analyzed with artificial intelligence and provide a therapy suggestion. Based on this information, a specialist physician (m/f/d) makes a therapy decision. - After the treatment intervention, the patient is provided with an AI-based messaging system. This system notifies the physician if the patient has complications or if the rehabilitation process seems abnormal. - No answer |
| **10. What level of error do you think is acceptable for AI-based systems used in diagnosis or treatment decisions for orthopedic conditions?**  According to the average competence ...   - ... a resident physician (m/f/d) (professional experience < 6 years) - ... of a board certified orthopedist (m/f/d) (professional experience approx. 5 - 10 years) - ... of an attending physician (m/f/d) (professional experience approx. 10 - 15 years) - ... a recognized expert (m/f/d) (professional experience > 15 years) - ... no answer |
| **11. For which of the following do you see the greatest potential benefits in using AI systems in patient care? (Multiple choice)**   - Improved prevention - Improved accuracy in diagnostics - Improved consistency in diagnostics and treatment decisions - Improved prediction of individual risks and outcomes - Improved prediction of disease progression - Time saving and higher accuracy in radiological diagnostics - Time saving in radiographic surgery - More individual treatment - Less expensive treatment - More precise and minimally invasive surgical techniques - Improved remote monitoring of patients - Reduction of postoperative complications - None of the above - No answer |
| **12. For which of the following do you see the greatest potential benefits in using AI systems activities outside direct patient care? (Multiple choice)**   - Improved and simplified documentation - Improved and simplified billing - Reduction of time spent by (specialist) physicians on monotonous and administrative tasks - Improved and more targeted access to subject-specific literature - Partial replacement of deficient administrative staff - None of the above - No answer |
| **13. For which of the following do you see the greatest potential drawbacks to using artificial intelligence systems in your field? (Multiple choice)**   - The treatment of orthopedic pathologies is too manual for AI - The treatment of orthopedic pathologies is too complex for AI - Difficulty to understand how the AI makes decisions (black box effect) - Declining skills of the next generation due to dependence on AI systems - Lack of ability to consider the emotional well-being of the patient - Lack of acceptance by patients - Ethical and liability issues in the event of complications - Risks for patient data (data security and data protection) - Undue influence of technology companies from outside of the medical field - Necessity of additional data collection and documentation - Additional costs - Reduction of medical orthopedic personnel - No answer |
| **14. In terms of the educational aspect, should ... (Multiple choice)**   - … AI be a part of medical school? - … AI be a part of residency training? - … AI be a part of educational courses? - … there is better information about the application areas of AI in orthopedics at orthopedic professional societies? - No answer |

***Supplementary table 1: Survey Questionnaire****. English translation of the questionnaire used in the survey. The survey was originally designed and distributed in German. Abbreviations: AI, artificial intelligence.*
