# Supplementary material for: Orthopaedic surgeons display a positive outlook towards artificial intelligence: A survey among members of the AGA Society for Arthroscopy and Joint Surgery
Source: J Exp Orthop. 2024 Jul 6;11(3):e12080. doi: 10.1002/jeo2.12080 (PMC11227606; doi:10.1002/jeo2.12080)
Supplement: Supplementary file 2 — Supporting information. [file JEO2-11-e12080-s004.docx]

| **Experience level** | **<5 years (%)** | **5-10 years (%)** | **11-15 years (%)** | **> 15 years (%)** | **p-value** |
| --- | --- | --- | --- | --- | --- |
| **How would you rate your knowledge of AI in medicine in general?** | | | | |  |
| *Expert knowledge* | 1 (2.4%) | 1 (1.6%) | 0 (0.0%) | **6 (3.1%)** | 0.021* |
| *Above average knowledge* | 6 (14.6%) | 3 (4.7%) | **12 (19.7%)** | 19 (9.8%) |  |
| *Average knowledge* | 13 (31.7%) | 26 (40.6%) | **29 (47.5%)** | 89 (46.1%) |  |
| *Rudimentary knowledge* | 16 (39.0%) | **32 (50.0%)** | 15 (24.6%) | 73 (37.8%) |  |
| *No knowledge* | **5 (12.2%)** | 2 (3.1%) | 5 (8.2%) | 6 (3.1%) |  |
| **Do you think AI will have a noticeable impact on joint surgery in the future, and if so, how long will it take?** | | | | | |
| *Never* | 0 (0.0%) | 1 (1.6%) | 0 (0.0%) | 4 (2.1%) | n.s. |
| *0-5 years* | 9 (22.5%) | 19 (30.2%) | 14 (23.0%) | 44 (22.8%) |  |
| *5-10 years* | 20 (50.0%) | 31 (49.2%) | 31 (50.8%) | 112 (58.0%) |  |
| *11-20 years* | 9 (22.5%) | 12 (19.0%) | 11 (18.0%) | 20 (10.4%) |  |
| *> 20 years* | 2 (5.0%) | 0 (0.0%) | 4 (6.6%) | 6 (3.1%) |  |
| *No answer* | 0 (0.0%) | 0 (0.0%) | 1 (1.6%) | 7 (3.6%) |  |
| **What level of error do you think is acceptable for AI-based systems used in diagnosis or treatment decisions for orthopaedic conditions?** | | | | | |
| *... a resident physician* | 10 (24.4%) | 8 (12.5%) | 12 (19.7%) | 23 (12.0%) | n.s. |
| *... of a board certified orthopedist* | 10 (24.4%) | 22 (34.4%) | 14 (23.0%) | 36 (18.8%) |  |
| *... of an attending physician* | 7 (17.1%) | 10 (15.6%) | 12 (19.7%) | 52 (27.1%) |  |
| *... a recognized expert in the field* | 12 (29.3%) | 16 (25.0%) | 15 (24.6%) | 56 (29.2%) |  |
| *... no answer* | 2 (4.9%) | 8 (12.5%) | 8 (13.1%) | 25 (13.0%) |  |

***Supplementary table 2:*** *Subgroup analysis of subjective knowledge of AI, anticipated impact of AI in the future, and acceptable level of error based on level of professional experience.* ***Bold*** *formatting is utilised in the statistically significant comparisons and indicates the most common answer in the respective line. Abbreviations: AI, artificial intelligence.*
