# Supplementary material for: Orthopaedic surgeons display a positive outlook towards artificial intelligence: A survey among members of the AGA Society for Arthroscopy and Joint Surgery
Source: J Exp Orthop. 2024 Jul 6;11(3):e12080. doi: 10.1002/jeo2.12080 (PMC11227606; doi:10.1002/jeo2.12080)
Supplement: Supplementary file 3 — Supporting information. [file JEO2-11-e12080-s002.docx]

| **Practice setting** | **University hospital** | **Academic teaching hospital** | **Non-academic hospital** | **Private practice** | **p-value** |
| --- | --- | --- | --- | --- | --- |
| **How would you rate your knowledge of AI in medicine in general?** | | | | |  |
| *Expert knowledge* | 2 (3.2%) | 0 (0.0%) | 2 (3.4%) | 4 (2.7%) | n.s. |
| *Above average knowledge* | 6 (9.7%) | 11 (12.5%) | 7 (12.1%) | 15 (10.3%) |  |
| *Average knowledge* | 28 (45.2%) | 42 (47.7%) | 23 (39.7%) | 62 (42.5%) |  |
| *Rudimentary knowledge* | 22 (35.5%) | 31 (35.2%) | 22 (37.9%) | 59 (40.4%) |  |
| *No knowledge* | 4 (6.5%) | 4 (4.5%) | 4 (6.9%) | 6 (4.1%) |  |
| **Do you think AI will have a noticeable impact on joint surgery in the future, and if so, how long will it take?** | | | | | |
| *Never* | 0 (0.0%) | 0 (0.0%) | 1 (1.8%) | 4 (2.7%) | n.s. |
| *0-5 years* | 18 (29.5%) | 16 (18.2%) | 14 (24.6%) | 37 (25.3%) |  |
| *5-10 years* | 25 (41.0%) | 55 (62.5%) | 28 (49.1%) | 82 (56.2%) |  |
| *11-20 years* | 15 (24.6%) | 13 (14.8%) | 11 (19.3%) | 13 (8.9%) |  |
| *> 20 years* | 2 (3.3%) | 2 (2.3%) | 3 (5.3%) | 5 (3.4%) |  |
| *No answer* | 1 (1.6%) | 2 (2.3%) | 0 (0.0%) | 5 (3.4%) |  |
| **What level of error do you think is acceptable for AI-based systems used in diagnosis or treatment decisions for orthopaedic conditions?** | | | | | |
| *... a resident physician* | 9 (14.5%) | **20 (22.7%)** | 7 (12.1%) | 16 (11.0%) | 0.004* |
| *... of a board certified orthopedic surgeon* | **21 (33.9%)** | 20 (22.7%) | 13 (22.4%) | 27 (18.6%) |  |
| *... of an attending physician* | 13 (21.0%) | 17 (19.3%) | 16 (27.6%) | 34 (23.4%) |  |
| *... a recognized expert in the field* | 17 (27.4%) | 17 (19.3%) | **21 (36.2%)** | **42 (29.0%)** |  |
| *... no answer* | 2 (3.2%) | 14 (15.9%) | 1 (1.7%) | 26 (17.9%) |  |

***Supplementary table 3:*** *Subgroup analysis of subjective knowledge of AI, anticipated impact of AI in the future, and acceptable level of error based on the practice setting.* ***Bold*** *formatting is utilised in the statistically significant comparisons and indicates the most common answer in the respective column. Abbreviations: AI, artificial intelligence.*
