# Supplementary material for: Orthopaedic surgeons display a positive outlook towards artificial intelligence: A survey among members of the AGA Society for Arthroscopy and Joint Surgery
Source: J Exp Orthop. 2024 Jul 6;11(3):e12080. doi: 10.1002/jeo2.12080 (PMC11227606; doi:10.1002/jeo2.12080)
Supplement: Supplementary file 4 — Supporting information. [file JEO2-11-e12080-s001.docx]

| **Familiar with digital applications** | **no** | **yes** | **p-value** |
| --- | --- | --- | --- |
| **How would you rate your knowledge of AI in medicine in general?** | | | |
| *Expert knowledge* | 2 (3.4%) | 6 (2.0%) | <.001 |
| *Above average knowledge* | 3 (5.2%) | 37 (12.3%) |  |
| *Average knowledge* | 14 (24.1%) | 143 (47.4%) |  |
| *Rudimentary knowledge* | 31 (53.4%) | 106 (35.1%) |  |
| *No knowledge* | 8 (13.8%) | 10 (3.3%) |  |
| **Do you think AI will have a substantial impact in the future, and if so, how long will it take?** | | | |
| *Never* | 3 (5.2%) | 2 (0.7%) | <.001 |
| *0-5 years* | 9 (15.5%) | 77 (25.7%) |  |
| *5-10 years* | 28 (48.3%) | 167 (55.7%) |  |
| *11-20 years* | 11 (19.0%) | 41 (13.7%) |  |
| *> 20 years* | 2 (3.4%) | 10 (3.3%) |  |
| *No answer* | 5 (8.6%) | 3 (1.0%) |  |
| **What level of error do you think is acceptable for AI-based systems used in diagnosis or treatment decisions for orthopedic conditions?** | | | |
| *... a resident physician* | 10 (17.5%) | 43 (14.2%) | 0.05 |
| *... of a board certified orthopedist* | 13 (22.8%) | 69 (22.8%) |  |
| *... of an attending physician* | 9 (15.8%) | 72 (23.8%) |  |
| *... a recognized expert in the field* | 12 (21.1%) | 88 (29.1%) |  |
| *... no answer* | 13 (22.8%) | 30 (9.9%) |  |

***Supplementary table 4:*** *Subgroup analysis of subjective knowledge of AI, anticipated impact of AI in the future, and acceptable level of error based on familiarity with digital applications not related to AI.* ***Bold*** *formatting is utilised in the statistically significant comparisons an indicates the most common answer in the respective column. Abbreviations: AI, artificial intelligence*.
